# Land Use as a Driver of Patterns of Rodenticide Exposure in Modeled Kit Fox Populations

**DOI:** 10.1371/journal.pone.0133351

**Published:** 2015-08-05

**Authors:** Theresa M. Nogeire, Joshua J. Lawler, Nathan H. Schumaker, Brian L. Cypher, Scott E. Phillips

**Affiliations:** 1 School of Environmental and Forest Sciences, University of Washington, Seattle, Washington, United States of America; 2 Western Ecology Division, U.S. Environmental Protection Agency, Corvallis, Oregon, United States of America; 3 California State University Stanislaus, Endangered Species Recovery Program, Turlock, California, United States of America; University of Southern California, UNITED STATES

## Abstract

Although rodenticides are increasingly regulated, they nonetheless cause poisonings in many non-target wildlife species. Second-generation anticoagulant rodenticide use is common in agricultural and residential landscapes. Here, we use an individual-based population model to assess potential population-wide effects of rodenticide exposures on the endangered San Joaquin kit fox (*Vulpes macrotis mutica*). We estimate likelihood of rodenticide exposure across the species range for each land cover type based on a database of reported pesticide use and literature. Using a spatially-explicit population model, we find that 36% of modeled kit foxes are likely exposed, resulting in a 7-18% decline in the range-wide modeled kit fox population that can be linked to rodenticide use. Exposures of kit foxes in low-density developed areas accounted for 70% of the population-wide exposures to rodenticides. We conclude that exposures of non-target kit foxes could be greatly mitigated by reducing the use of second-generation anticoagulant rodenticides in low-density developed areas near vulnerable populations.

## Introduction

As their habitats become scarcer and more fragmented, many populations of animals increasingly rely on human-dominated landscapes during all or part of their life cycles [1–4]. In California, where only 25% of natural land cover remains [5], agricultural lands provide some habitat value to nearly half of the state’s terrestrial vertebrate species [6]. Similarly, many wildlife species use lands with other types of human disturbances [7], such as low-density housing or energy developments. Not surprisingly, animals in these human-dominated landscapes often face additional threats such as conflicts with humans [8], have higher mortalities and lower fitness [9], and may exist at lower densities than those inhabiting more natural landscapes [10].

Second-generation anticoagulant rodenticides (SGARs) are commonly used in agricultural [11] and exurban [12,13] landscapes, and have occasionally been found to be used illegally in wildlands [14]. Legal SGAR use is confined to applications in and around the perimeter of buildings [15], and both the U.S. Environmental Protection Agency (EPA) and the California Department of Pesticide Regulation have recently changed regulations with the purpose of reducing non-target wildlife exposure. Nonetheless, the use of SGARs may become even more widespread in response to projected climate change-induced pest outbreaks [16].

Rodenticides such as brodifacoum, bromadiolone, difenacoum, and difethialone are intended to poison rodents, but widespread exposure to these compounds is found in carnivorous and omnivorous mammals and birds. In California, these affected mammals include San Joaquin kit foxes (*Vulpes macrotis mutica*), bobcats (*Lynx rufus*), cougars (*Puma concolor*), and fishers (*Pekania pennanti*) [14,15]. Exposure to SGARs has also been found in raptors in San Diego County [17], raptors in New York [15], red kites (*Milvus milvus*) in France [18], 62 vertebrate species in Spain [19], birds, amphibians, and reptiles in New Zealand [20,21], and rodents and raptors in the UK [14,22,23]. The U.S. EPA states that they believe that exposure of non-target wildlife is occurring wherever these SGARs are used, based on the widespread exposure that is found when surveys of non-target populations are conducted [15]. Exposure of non-target wildlife occurs via both direct consumption of bait or, in the case of carnivorous animals, by secondary exposure via the consumption of contaminated prey items [15]. Secondary exposure is of particular concern because these compounds are persistent in body tissues, and time-to-death after initial consumption is 5–7 days, potentially allowing animals to consume many times the lethal dose and resulting in death of individuals [15]. Non-target exposures to SGARs and other pesticides are of particular concern when they affect threatened or endangered species such as red kites in France [18] or Pacific fishers in California [24].

One such at-risk species is the San Joaquin kit fox, a small desert fox that persists primarily around the perimeter of the San Joaquin Valley, a major agricultural area in central California extending from the San Joaquin delta to the Tehachapi Mountains. The San Joaquin kit fox (hereafter “kit fox”) is an endangered subspecies that eats primarily kangaroo rats (*Dipodomys spp*) where their ranges overlap, or a variety of other small animals, including voles (subfamily Arvicolinae), ground squirrels (subfamily Xerinae), rats (*Rattus spp*), mice (*Mus musculus*, family Cricetidae, and family Heteromyidae), rabbits (family Leporidae), gophers (family Geomyidae), and insects (class Insecta) [25]. The current population of the kit fox is estimated to be fewer than 3600 individuals [26]. Kit fox exposure to SGARs has been repeatedly documented (e.g., [26–28]), and is thought to result from the consumption of contaminated rodents (either target or non-target) or as a result of eating bait directly [29].

Simple non-spatial models suggest that SGAR exposure could result in population-level effects for kit foxes [30], but spatially-explicit models are also needed that can scale individual effects up to population levels while accounting for spatial variation in exposure rates [31,32]. Spatial models can predict where kit foxes are likely exposed to rodenticides, and can thus help regulators target mitigation efforts through education, regulation, or enforcement. Using land-cover mapping and estimated rodenticide use levels in each land-cover category, we created a map of projected kit fox exposure probabilities. We then used a spatially explicit, individual-based population model, which included life history traits and kit fox ecology, to measure the impact of SGAR exposure on modeled kit foxes across their range. This detailed, mechanistic population model also allowed us to evaluate management-relevant patterns of exposure.

## Methods

### Simulating kit fox populations

We simulated individual kit foxes across their range using HexSim [33], a computer modeling platform for constructing spatially explicit population models. Our model integrated life history traits, repeated exposures to rodenticides, and spatial data layers describing habitat and locations of likely exposures. We modeled female kit foxes using yearly time steps in which each individual had the potential to disperse, establish a home range, acquire resources from their habitat, reproduce, accumulate rodenticide exposures, and die.

**Fig 1:**
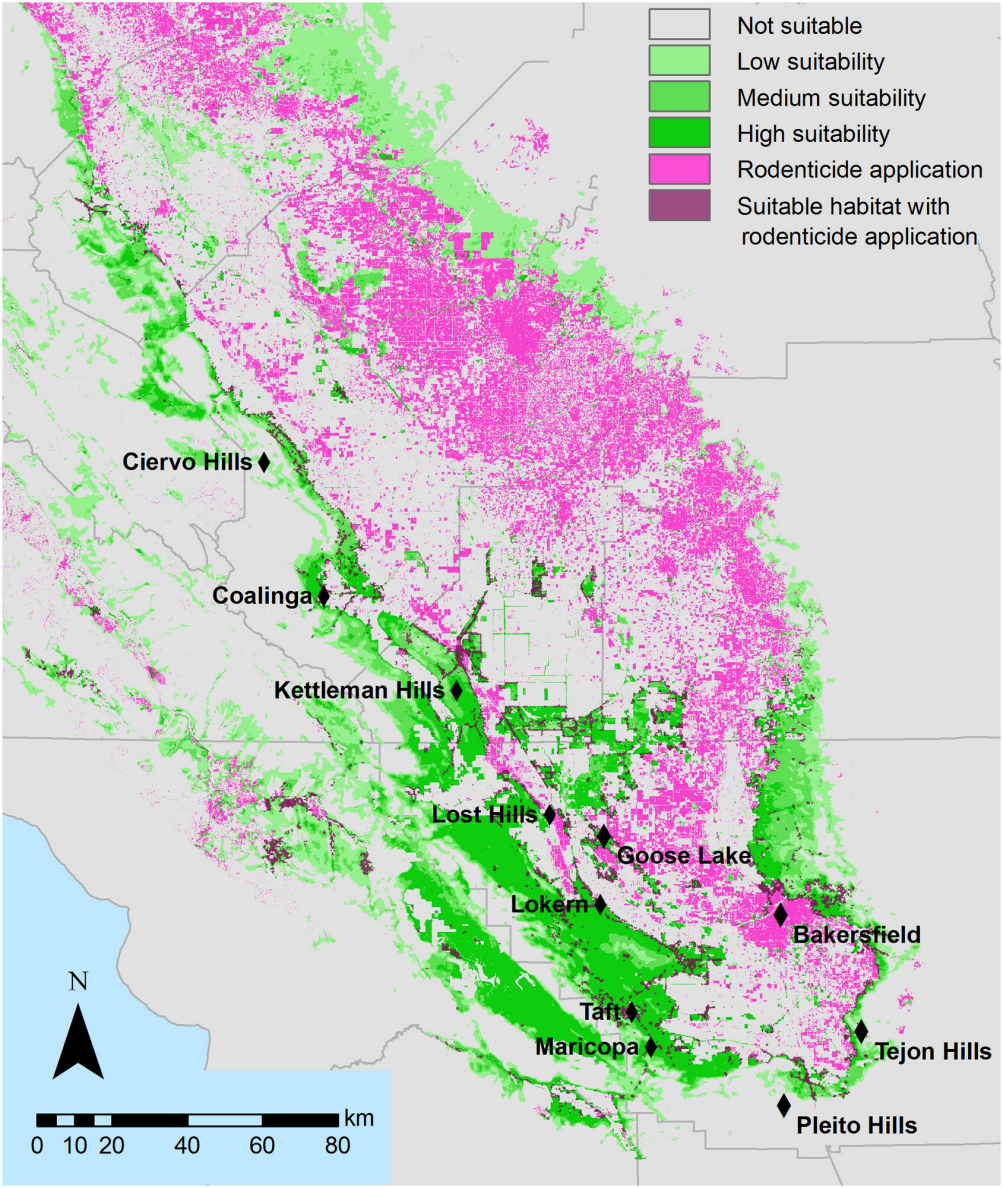
Habitat suitability and rodenticide use. San Joaquin kit fox suitable habitat (green) and rodenticide use within the kit fox range (purple). Rodenticide use is defined as 30 x 30 m pixels which included land use where rodenticides are commonly used. Not suitable is defined as suitability = 0, low suitability is 2–4, medium suitability is 4–6.5, and high suitability is 6.5–8. Habitat suitability is from [26] and rodenticide use was defined as described in the methods. The map was created in ArcMap 10.2.

We used a map of habitat suitability developed by Cypher et al. [26] to inform home range establishment, resource accumulation, and dispersal. The suitability map was based on land-cover data from the California Department of Water Resources Land Use Survey, California Gap Analysis Program, National Wetlands Inventory, aerial photography, vegetation density, terrain ruggedness, and expert opinion (see S1 Table). The suitability map includes 4,239 km^2^ of high suitability (suitability score > 90) habitat and 9,430 km^2^ of medium suitability (> 75) habitat (Fig 1a) throughout the kit fox range. Approximately two thirds of this habitat is fragmented: only 2,550 km^2^ of high-suitability habitat (60%) and 6,498 km^2^ of medium suitability habitat (69%) occurred in patches of greater than 50 km^2^. We tessellated the habitat suitability map using a grid composed of 14-ha hexagons, and each hexagon derived a habitat “score” from the underlying habitat map. Habitat suitability was presumed to be the same for breeding, movement, dispersal, and exploration. Kit foxes were precluded from incorporating unsuitable habitat in home ranges, but were able to move through and explore these areas.

Simulated kit foxes assembled home ranges based on local habitat suitability, with range size inversely related to habitat suitability [34,35]. Kit foxes aimed to acquire a home range with a target score corresponding to the observed 544 ha home range size in the most suitable habitat [26]. Modeled home ranges varied in size from 170 ha to 1000 ha. Kit foxes were assigned to a resource class depending on the quality of the habitat in their acquired home range. The resource class then influenced rates of kit fox survival, with kit foxes being able to survive if they accumulated at least 30% of the target habitat score (Table 1). Reproductive success in kit foxes varies greatly. Field study estimates range from 67% to 100% success in the best habitats and as low as 20% success in poor habitats [29]. As in Haight et al. [36], we represented adult average reproductive success with a mean value of 61%. Individual values were linearly scaled depending on resource class, and ranged from 0 to 100% success (Table 1). Modeled adult kit foxes that reproduced produced 1–3 female pups, with a normal distribution around a mean of 1.9 females (derived from field-based estimates from [29] and a standard deviation of 0.95 females), which results in approximately 17% of litters with 3 female pups. Yearling kit foxes occasionally breed, but their reproductive success rates and litter sizes have not been well studied. We estimated yearling reproductive success using data from a single study in which 22 yearlings reproduced with one-third the success rate of adults in the same study area [37]. Given that the yearling foxes in the study presumably used lower-quality habitat, on average, than adults, and given that our own models scale reproductive rates based on habitat quality, we estimated yearling reproductive success to be half that of adults in any given resource class. We believe this represents an optimistic estimate of yearling reproductive success.

**Table 1:** Survival and reproductive success rates for modeled kit foxes.

Percent of target habitat score accumulated (lower bound of resource class)	Juvenile survival	Adult and subadult survival	Subadult reproductive success	Adult reproductive success
0	0	0	0	0
10	0	0	0	0
20	0	0	0	0
30	0.2	0.4	0.15	0.3
40	0.3	0.6	0.2	0.4
50	0.45	0.7	0.25	0.5
60	0.55	0.8	0.3	0.6
70	0.65	0.85	0.35	0.7
80	0.75	0.9	0.4	0.8
90	0.85	0.95	0.45	0.9

Rates scaled according to the quality of the habitat in each kit fox’s range (represented as percent of target habitat accumulated).

Juveniles and adults without ranges searched for a home range across 30 km^2^ outside of their natal range, using HexSim’s ‘adaptive’ exploration algorithm [33]. In wild populations, kit foxes without established home ranges are able to pass through and hunt within conspecific home ranges due to lack of absolute territoriality [38–40], and we modeled this by allowing such individuals to survive by using up to half of the habitat resources (“score”) in another kit fox’s range. These individuals, however, were subject to 40% lower survival rates. We used an estimated actual population size of 2500 females based on initial population estimates to initialize the model. The 2500 individuals were distributed across the best habitat throughout the kit foxes’ range.

### Rodenticide exposure

To assess the potential effects of rodenticides on kit fox populations we mapped areas of likely exposure to SGARs, applied a range of estimated SGAR-induced mortality rates to individuals that encountered rodenticides, and measured changes in kit fox population size and distribution. To map exposure we used a state agricultural database [11], surveys of residents [13], and liver tests of 68 opportunistically found kit foxes in and around Bakersfield [41,42] to identify land-use types in which SGARs are used. We assigned scores that described the relative likelihood of rodenticide exposure within different land-cover types. Land-cover types with high levels of rodenticide use, including confined animal agriculture, semi-agricultural, or low-density development (see S2 Table for definitions), were assigned a score of “2” [11]. We included low-density residential areas in the high-likelihood zone for two reasons. First, consumers and professional applicators frequently use SGARs for rodent control in such areas [43–46], and second, McMillin et al. [42] documented exposure in kit foxes in these areas. Land-cover types with intermediate levels of rodenticide use, including urban lands and orchards [11,13,47], were given a score of “1”. Areas where SGARs are generally not used, including natural land-cover types, farmland, or grazing lands [11,42], were assigned a score of “0” (see S2 Table for details).

For each kit fox with a home range, the score for likelihood of exposure for that home range in each year was used to assign the individual to an overall exposure class. For each kit fox without a home range, the score for the explored area was used. Kit foxes that encountered rodenticides were classified into groups of low, medium or high exposure. Without clinical evidence to support the exposure levels, we set likelihood of exposure score thresholds so that 1/3 of the exposed animals would fall into each exposure class. Kit foxes born to exposed mothers were placed in the same exposure class as the mother. Exposure did not persist between years. Kit foxes of all age classes were subjected to additional mortality according to their rodenticide exposure class and exposure scenario (see Table 2).

**Table 2:** Additional mortality suffered by kit foxes with varying amounts of rodenticide exposure.

Scenario	None	Low	Medium	High
*Range of exposure scores*	*0*	*1–2*	*3–6*	*7+*
No effect	0	0%	0%	0%
Moderate effect	0	2%	6%	25%
High effect	0	9%	28%	67%

All estimates adapted from Giddings & Warren-Hicks [30]. For example, a kit fox with a high level of exposure suffers 25% additional mortality in our ‘moderate-effect’ scenario.

Although no studies have directly quantified the lethal dose of SGARs for kit foxes, Giddings and Warren-Hicks [30] were able to identify the range of mortality effects likely experienced by kit fox populations exposed to brodifacoum (the most commonly used SGAR). They estimated the median lethal dose sufficient to kill 50% of a population (LD50), and generated dose-response curves, basing curve shapes on a literature review of responses to brodifacoum observed in dogs, feral pigs, guinea pigs, mice, rabbits, rats, sheep and wallaby. Based on their estimate that 1–2.5% of prey items are exposed to brodifacoum, they then simulated probable effects on mortality of individual kit foxes. Our mortality rates (Table 2) were based on these mortality risk estimates. We used the upper bound of the Giddings and Warren-Hicks [30] estimate because those authors concluded that even a 2.5% exposure rate is likely an underestimate. We used these rates, based on exposure to brodifacoum, for all SGAR exposure, because more specific data were not available for other types of SGARs.

We considered three scenarios of exposure effects based on mortality rates from Giddings and Warren-Hicks’ models, with each scenario being run 100 times, based on a leveling off of variance. Our *high-effect scenario* (S1 File) used 10% exceedance values (a 10% chance that the real mortality levels exceed the given value), our *moderate-effect scenario* (S2 File) used 50% exceedance values, and our *no-effect scenario* (S3 File) had zero additional mortality from exposure to rodenticides. We then consider two *regulated* scenarios, in which exposure was eliminated from low-density developed lands (to simulate a situation in which rodenticides are no longer used in these lands) but remains the same in other land-cover types. Finally, we multiplied the map of modeled occupancy by the map of exposure likelihood to determine the relative contribution of each land-cover type to exposure.

### Sensitivity analyses

For our model parameters we were able to draw upon the considerable data available from the literature, as cited throughout the manuscript, for this well-studied species. Nonetheless, we still needed to make assumptions and estimates. We tested the impact of at least a 10% increase or decrease in starting population, dispersal range, floater mortality, and threshold for exposure classes, with 50 replicates of each scenario. We also tested the categorization of “Urban” lands with a likelihood of exposure score of “2” instead of “1”, again with 50 replicates. Finally, we tested the sensitivity of the parameter defining additional mortality suffered by exposed foxes. We ran 100 replicates of seven scenarios in which we adjusted the additional mortality to 50% below our moderate scenario, 10% below our moderate scenario, halfway between our moderate and high scenarios, 10% above our high scenario, 50% above our high scenario, 90% above our high scenario, and complete mortality (all exposed foxes die).

## Results

**Fig 2:**
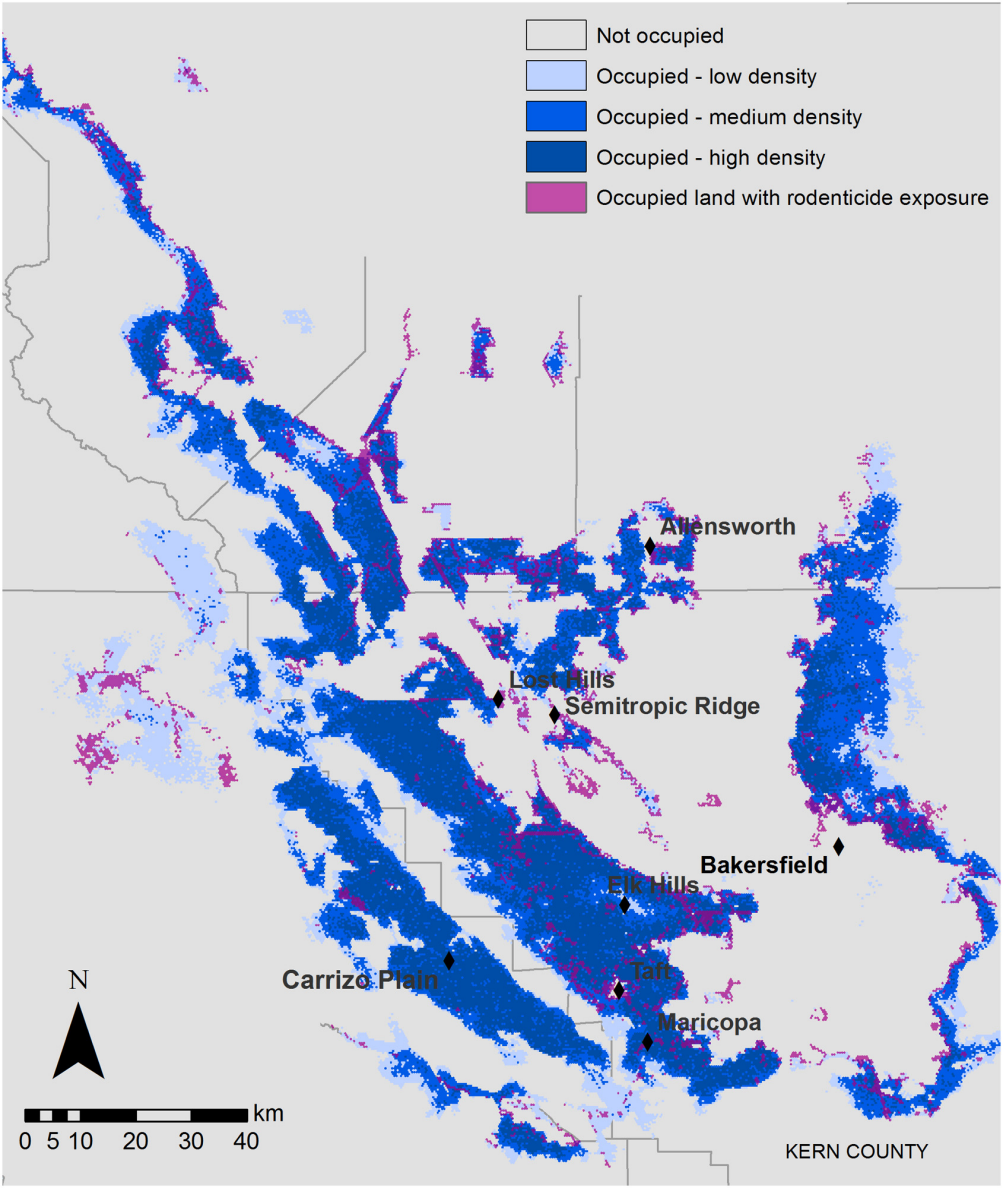
San Joaquin kit fox modeled occupancy overlap with areas of rodenticide use. Source of mapped data are model outputs, created in ArcMap 10.2.

Roughly 12% (491 km^2^) of the most suitable, occupied kit fox habitat and 4.3% (225 km^2^) of occupied habitat of moderate suitability was predicted to have rodenticide use (Fig 1). As expected, rodenticide exposure occurred primarily around the edges of kit fox habitat, and in areas where habitat was more fragmented (primarily by agriculture). Highly affected patches occurred around the Semitropic Ridge, Allensworth Natural Area, Lost Hills, and near the cities of Bakersfield, Taft and Maricopa, which host urban kit fox populations (Fig 2). Unaffected habitat patches were found in the Carrizo Plain and in western Kern County.

Thirty-six percent of our modeled kit foxes were exposed to rodenticides. Low-density development accounted for 70% of the exposures, followed by orchards (17%) and urban areas (6.8%). Although confined animal agriculture and semi-agricultural lands had the highest likelihood of exposure, these land-cover types were not commonly occupied by kit foxes and were thus responsible for a low percentage of kit fox exposure (0.48% and 5.9%, respectively). Modeled exposures occurred primarily around the edges of core habitat blocks throughout the southern part of the kit foxes’ range (Fig 2). A large area of exposures occurred in the Elk Hills, near the cities of Maricopa and Taft, and the surrounding areas. Modeled exposure did not occur in the Carrizo Plain.

On average, our no-effect scenario yielded a population of 2075 female kit foxes (i.e., 4150 total kit foxes), of which approximately 77% were breeding adults. In our model, mean home range size was 3.76 km^2^ (1.81–9.95 km^2^, stdev = 1.45, for all home ranges and 100 replicates). The distribution of modeled kit foxes, shown in S1 Fig, is consistent with areas in which kit foxes are known to occur with the exception of the area north of Bakersfield, where kit foxes have not been reliably sighted [48].

**Fig 3:**
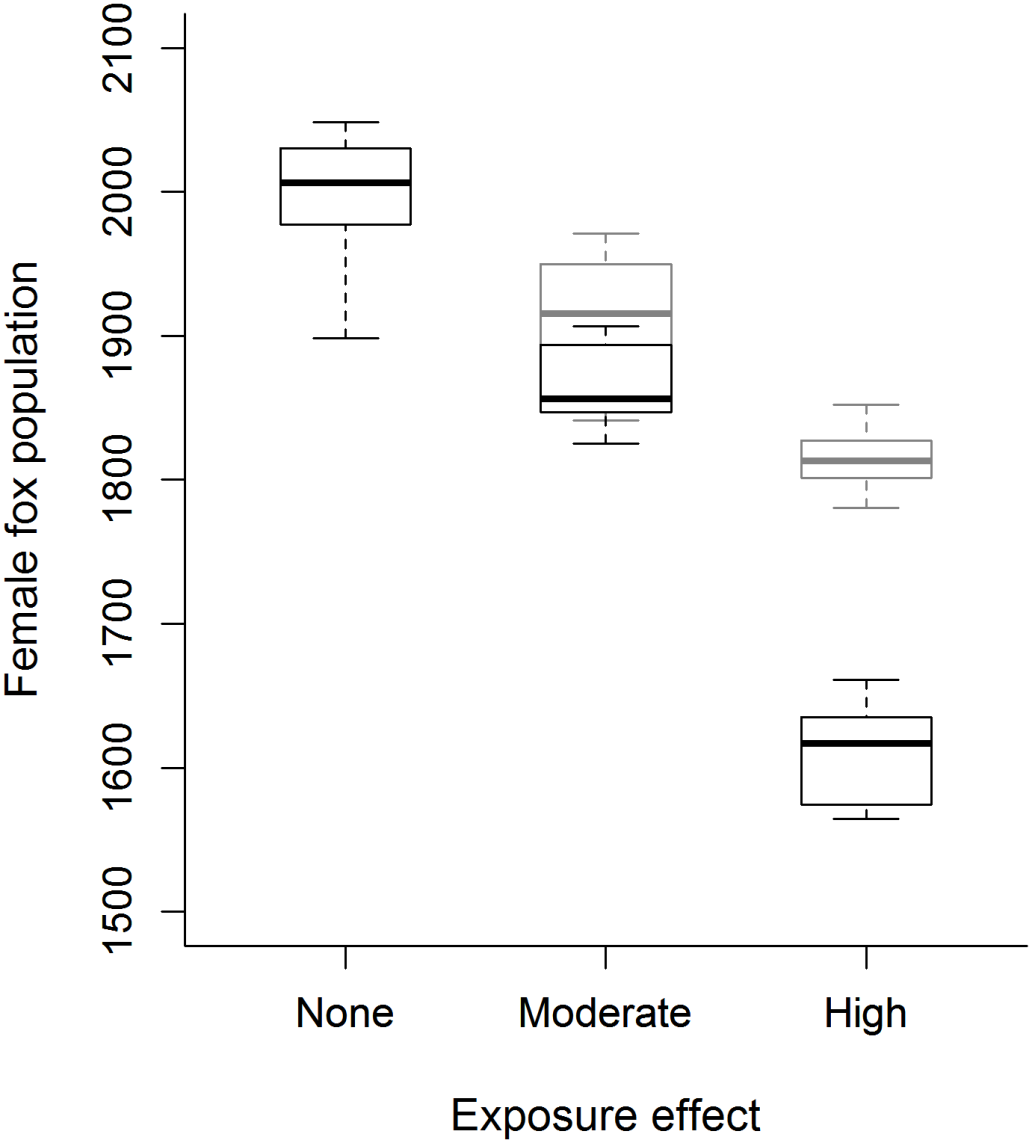
Impact of rodenticide application on kit fox population. Boxplot of impact of rodenticide application on female kit fox populations across 100 replicates. Black boxes indicate no-, moderate-, and high-effect scenarios. Grey boxes indicate moderate- and high-effect scenarios with no rodenticides in low-intensity development land cover class.

SGAR exposure affected both the overall simulated kit fox population size (Fig 3) and the distribution of the population (S1A and S1B File). Modeled rodenticide exposure under the moderate-effect scenario caused a 7% population decline relative to the no-effect scenario (Fig 3). Modeled kit fox populations in this scenario were reduced from the area that is directly to the east of Bakersfield, south to the Tejon Ranch and west to the Pleito Hills, in the Buttonwillow Ridge, near Goose Lake, and in the Kettleman Hills. The modeled population declined by 18% under the high-effect scenario (Fig 3), and populations were further reduced in the areas described in the moderate-effect scenario as well as in the Ciervo Hills. In the high-effect scenario, the Pleito Hills/Tejon Ranch population became separated from the population east of Bakersfield. The regulated scenario, where SGARs were not used in low-density developed lands, produced an increase in the population in both the moderate-effect (4.5% increase) and high-effect (9.3% increase) scenarios (Fig 3).

### Sensitivity analyses

Changing the size of the area explored by dispersers resulted in the greatest change to the total population size (a 10% decrease in the parameter value resulted in a 4% decrease in total population size; a 10% increase in the parameter value resulted in a 2% increase in total population size), followed by mortality for kit foxes with no home range (1%; 0%), the threshold of the different exposure classes (1%; 0%), and the starting population size (1%; 0%) (Table 3). Percent of population exposed, locations of exposures, and land covers with the greatest impact were not sensitive to any of the tested parameters.

**Table 3:** Sensitivity analyses: percent change in total female kit fox population relative to baseline when key parameters were increased and decreased 10%.

Parameter	- 10%	+ 10%
Starting population	0%	0%
Dispersal range	-4%	2%
Floater mortality	-2%	0%
Threshold for exposure classes	-1%	0%

The impact of rodenticides on the total population was not sensitive to the above changes in parameters. Each resulted in a maximum 1% difference between the no-effect and moderate-effect or no-effect and high-effect scenarios. However, the impact of rodenticides on the total population was, as expected, sensitive to the parameter defining additional mortality suffered by exposed foxes. The scenarios with mortality rates 50% below and 50% above our estimated rates would result in a 4–22% total population decline (compared to no effect), as opposed to the 7–18% decline that our model predicted for our estimated rates. S2 Fig shows the range of population sizes under the various scenarios.

We found little difference in the total population size when testing the categorization of Urban Lands with a likelihood of exposure score of “2” instead of “1” (the population decreased 1.1% with a score of “2” relative to the scenario with a score of “1”). This is unsurprising because the majority of the “urban” category consists of high density housing, which is characterized as low-quality habitat for foxes. These areas were thus rarely explored by modeled foxes, and changing their likelihood score resulted in few additional exposures or deaths.

## Discussion

Pesticides are known to negatively impact non-target species, and SGAR residues have been found in many non-target species [14,15,17,20,49], including the kit fox [41,42]. Our study is one of the first to examine how rodenticides affect an entire wildlife population across its range, and where, in a complex landscape, those effects might be most severe (but [49] also considers spatial impacts across a limited population). Our model predicted that 36% of kit foxes were likely exposed to SGARs, resulting in an estimated 7–18% reduction in the population depending on the mortality rate.

Ours is the first mechanistic population model of the entire San Joaquin kit fox population. The resulting estimates of total population numbers were similar to those produced by Cypher et al. [26], who extrapolated density estimates from survey sites to other suitable habitat using the same habitat map used in the present study. Our estimates ranged from 3300 (high effect of exposure) to 4150 (no effect of exposure), while Cypher et al. estimated 3600 kit foxes, but argued that this was likely an overestimate [26]. Not all suitable habitat was occupied by kit foxes in our model runs, which is consistent with the observation that some habitat patches were isolated and thus difficult for dispersers to reach, and a large proportion (66%) of habitat was fragmented and unlikely to support persistent kit fox populations. In addition, some habitat was periodically vacant even within substantial habitat patches, indicating that the modeled population size was limited by high mortality rates rather than quantity of habitat. This was supported by field studies that concluded that high mortality rates, due in large part to coyote predation, limit populations in suboptimal habitats [50].

Several assumptions and approximations likely impact our estimated percentage declines in the kit fox population as a result of rodenticide exposures. It was difficult to map all land-cover types where rodenticides were applied. For example, the NLCD category of Developed, Open Space included some types of lands where SGARs were likely used, such as golf courses, parks, and very low-density housing. However, the majority of lands in this category, which often included dirt roads, were not expected to have rodenticides, so this category was classified as “0” likelihood of exposure. Additionally, application of SGARs in low-density developments may have decreased due to regulations put in place in 2008, since the studies used to parameterize our model were conducted. Further, this study examined only SGAR usage, but kit foxes are also exposed to first-generation anticoagulant rodenticides (FGARs). FGARs may have less impact on kit foxes because they are not as persistent, leaving less time for the toxicant to affect higher trophic levels. However, FGARs are also more widely used in areas frequented by kit foxes, particularly in rangelands, where they are broadcast-baited in grains.

The mortality estimates we used are based on several assumptions which could have influenced our population estimates. First, we use brodifacoum-based toxicity estimates for all SGARs, although in reality other SGARs have lower toxicity (Eason et al [51], for example, summarize toxicity of several SGARs). We use these estimates because brodifacoum is the most commonly found SGAR in tested wildlife (some examples from carnivores in California include [12,14,42]) and because no other estimates were available. Second, Giddings and Warren-Hicks assume no illegal use of SGARs, although as they note, such misuse is probably very common, and misuse was also reported by surveyed residents in a separate study [45]. Field testing of other species supports the idea that our SGAR exposure estimates were likely conservative. In comparison, anticoagulant rodenticide exposure levels in southern California were 90% for bobcats [12] and 70% for all mammals and birds tested by California Department of Fish and Wildlife [15]. Third, the mortality estimates we applied in the areas of high likelihood of exposure are likely to be conservative. We based our estimates on the assumption that 2.5% of all rodents consumed by kit foxes across their range were exposed to rodenticides [30], but we only applied these estimates to areas where SGAR use was likely. Thus, the percentage of rodents exposed to SGARs in high-likelihood areas could be expected to be much higher than 2.5%. We would expect considerable variation in the percentage of rodents exposed even within 30-m pixels, because the percent of rodents that are exposed will vary based on their distance from the site of application. For example, at sites in close proximity to where SGARs were applied, Tosh, et al. [52] found 15–33% of target mice were exposed and Brakes and Smith [21] found an average exposure rate of 48.6% across three species of non-target rodents. Another study, focused on non-target rodent exposure, found that most of the rodents with SGAR residues were within 15 m of application sites [53]. Finally, although kit foxes are frequently observed near structures, we would expect variation in kit fox hunting preferences in relation to these structures.

Sublethal impacts of rodenticides, which we did not simulate, also have an indirect effect on mortality [15,21,30,44]. When not immediately fatal, SGAR exposure can still cause weakness, lack of coordination, rapid breathing, depression, severe abdominal pain, and loss of appetite [54], all of which could significantly reduce fitness. For example, SGARs have been implicated in an increased frequency of vehicle strikes in fishers [23] and increased mortality in bobcats due to a mange epidemic, where Riley et al. [12] hypothesize that mites benefited from reduced clotting caused by SGAR exposure. Other studies have been inconsistent in the degree of attribution of SGAR exposure to deaths in SGAR exposed birds and mammals. In some studies, it has been found that less than 10% of deaths in animals with SGAR residues were definitively attributed primarily to SGAR exposures [19,55–57], although another study found that it was “highly probable” that 50% of deaths in animals with SGAR residues were caused by the SGARs [15]. These inconsistent results further supports the need for further studies to determine what, if any, sublethal impacts are caused by SGARs. Some researchers also note that the number of confirmed cases likely underestimates the number of actual deaths caused by SGARs [19,21,55,56,58], primarily because of the difficulty in detecting microscopic hemorrhages.

### Spatial patterns and management implications

Although 36% of modeled kit foxes were exposed, the exposures took place on a relatively small portion (16%) of the landscape. Low-density development was the largest source of exposures of kit foxes to SGARs. Low-density development most commonly includes single-family housing units. In many instances, residents do not know which chemicals are used to control rodents on their properties, nor the mode of action of these chemicals. However, owners of these single-family units are generally interested in learning about the effects rodenticides have on of non-target species [46], suggesting that education programs could help reduce the impact of anticoagulant rodenticide usage. Kit foxes have also been enormously successful in some areas with low-density development. Estimates of survival and reproduction, for example, are higher within the city of Bakersfield than in wildland populations [29]. Urban populations are also becoming established in the cities of Taft and Maricopa. In these urban settings, rodenticide exposures could be offset by the positive impacts of fewer predators and a more steady food supply, and reducing exposure may therefore not be a high priority. Exposure may be more of a concern in low-density development that is near the natural habitat of kit foxes, and it is this exposure that has the greatest population-wide effect in our model. Successful enforcement of SGAR regulations and additional regulations or education discouraging their use in low-density developments within the kit fox range could also increase kit fox population numbers.

## Supporting Information

S1 Fig Mapped comparison of no-effect and high-effect scenarios. Suitable range-wide habitat with occupancy as modeled under the ‘no-effect’ scenario (S1A Fig) and ‘high-effect’ scenario (S1B Fig). Modeled kit foxes were concentrated in western Kern County and the Carrizo Plain, with populations continuing north along the western edge of the San Joaquin Valley, through the Lokern natural area and Kettleman Hills, north to the Panoche Hills. There were no persistent populations north of the San Luis Reservoir. The population also extended into the Valley around the Lost Hills, through the Semitropic Ridge natural area northeast to the Pixley National Wildlife Refuge. On the east side of the San Joaquin Valley, there was a large population of modeled kit foxes east of Bakersfield and north to the border of Kern County. South and east from Bakersfield, the population extended south through the Tejon Ranch and west to the Pleito Hills and the Wind Wolves Preserve. This distribution is entirely consistent with areas in which kit foxes are known to occur except the area north of Bakersfield, where kit foxes have not been reliably sighted [48]. The distribution of kit foxes in the high-effect scenario is more consistent with recent kit fox sightings, with less occupancy modeled north of Bakersfield, a gap in occupancy near northern Tejon, another gap between the Kettleman Hills and the Panoche area, and fewer kit foxes overall. Habitat suitability is from [25] and source of occupancy data are model outputs. The map was created in ArcMap 10.2.
(DOCX)

S2 Fig Fox population versus modeled mortality rate. The shaded portion of the plot indicates the range between our ‘moderate-effect’ and ‘high-effect’ scenarios. We also ran 100 replicates each of 8 additional scenarios ranging from no effect of exposure (0 additional mortality) to 100% mortality from exposure (all foxes that become exposed die). The x-axis is the mean mortality rate across the three classes of exposure.
(TIFF)

S1 File ‘High-effect’ HexSim scenario file. (XML)

S2 File ‘Moderate-effect’ HexSim scenario file. (XML)

S3 File ‘No-effect’ HexSim scenario file. (XML)

S1 Table Habitat suitability map. Assigned suitability values for land cover classes, scored from 0 (low) to 100 (high).
(DOCX)

S2 Table Land-cover classes, SGAR exposure score, and source of map data. (DOCX)
