# Supplementary material for: Land Use as a Driver of Patterns of Rodenticide Exposure in Modeled Kit Fox Populations
Source: PLoS One. 2015 Aug 5;10(8):e0133351. doi: 10.1371/journal.pone.0133351 (PMC4564287; doi:10.1371/journal.pone.0133351)
Supplement: S1 Fig — Suitable range-wide habitat with occupancy as modeled under the ‘no-effect’ scenario (S1A Fig) and ‘high-effect’ scenario (S1B Fig). Modeled kit foxes were concentrated in western Kern County and the Carrizo Plain, with populations continuing north along the western edge of the San Joaquin Valley, through the Lokern natural area and Kettleman Hills, north to the Panoche Hills. There were no persistent populations north of the San Luis Reservoir. The population also extended into the Valley around the Lost Hills, through the Semitropic Ridge natural area northeast to the Pixley National Wildlife Refuge. On the east side of the San Joaquin Valley, there was a large population of modeled kit foxes east of Bakersfield and north to the border of Kern County. South and east from Bakersfield, the population extended south through the Tejon Ranch and west to the Pleito Hills and the Wind Wolves Preserve. This distribution is entirely consistent with areas in which kit foxes are known to occur except the area north of Bakersfield, where kit foxes have not been reliably sighted [48]. The distribution of kit foxes in the high-effect scenario is more consistent with recent kit fox sightings, with less occupancy modeled north of Bakersfield, a gap in occupancy near northern Tejon, another gap between the Kettleman Hills and the Panoche area, and fewer kit foxes overall. Habitat suitability is from [25] and source of occupancy data are model outputs. The map was created in ArcMap 10.2. (DOCX) [file pone.0133351.s001.docx]

S1 Figure**. Mapped comparison of no effect and high effect scenarios.**


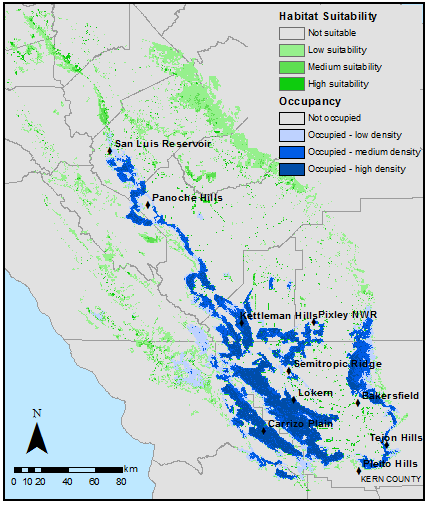

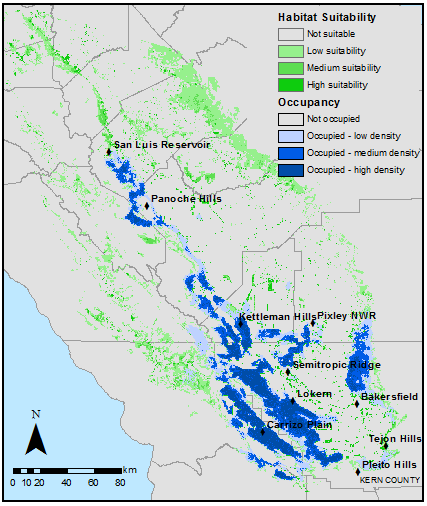


**B: High effect scenario**

**A: No effect scenario**
