# Supplementary figures and images for: Land Use as a Driver of Patterns of Rodenticide Exposure in Modeled Kit Fox Populations
Source: PLoS One. 2015 Aug 5;10(8):e0133351. doi: 10.1371/journal.pone.0133351 (PMC4564287; doi:10.1371/journal.pone.0133351)

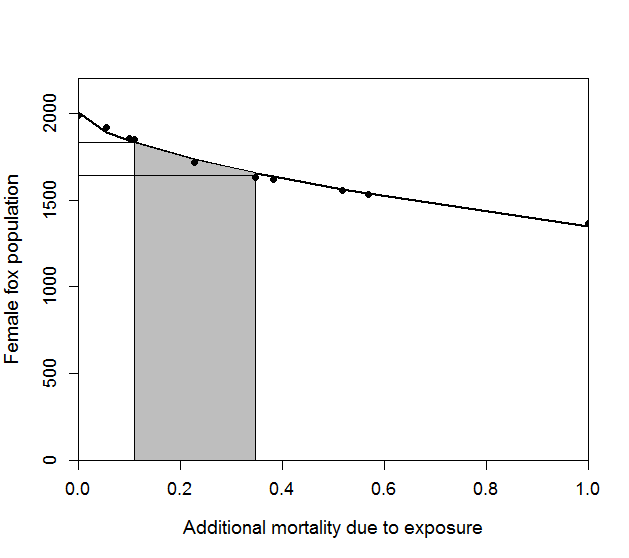

Supplement: S2 Fig — The shaded portion of the plot indicates the range between our ‘moderate-effect’ and ‘high-effect’ scenarios. We also ran 100 replicates each of 8 additional scenarios ranging from no effect of exposure (0 additional mortality) to 100% mortality from exposure (all foxes that become exposed die). The x-axis is the mean mortality rate across the three classes of exposure. (TIFF) [file pone.0133351.s002.tiff]
