# Supplementary material for: Land Use as a Driver of Patterns of Rodenticide Exposure in Modeled Kit Fox Populations
Source: PLoS One. 2015 Aug 5;10(8):e0133351. doi: 10.1371/journal.pone.0133351 (PMC4564287; doi:10.1371/journal.pone.0133351)
Supplement: S1 Table — Assigned suitability values for land cover classes, scored from 0 (low) to 100 (high). (DOCX) [file pone.0133351.s006.docx]

**S1 Table. Habitat suitability map.**

| **Land-use/land-cover class** | **Land-use/land-cover type** | **Habitat suitability value** |
| --- | --- | --- |
| Urban/Industrial | Oil field/Extractive | 65 |
|  | Urban | 20 |
|  | Urban commercial | 40 |
|  | Urban industrial | 40 |
|  | Urban landscaped | 60 |
|  | Urban residential | 10 |
|  | Urban vacant | 50 |
|  |  |  |
| Agriculture | Farmstead | 5 |
|  | Feed lot | 10 |
|  | Field crops | 10 |
|  | Grain/Pasture | 30 |
|  | Idled farmland | 50 |
|  | Orchard | 20 |
|  | Retired farmland | 75 |
|  | Rice | 5 |
|  | Vineyard | 10 |
|  |  |  |
| Rangeland | Desert scrub | 95 |
|  | Grassland | 90 |
|  | Grassland/ruderal | 75 |
|  | Heavy brush | 5 |
|  | Lowland scrub | 50 |
|  | Medium brush | 10 |
|  |  |  |
| Forested land | Brush and timber | 5 |
|  | Forest | 0 |
|  | Oak woodland | 15 |
|  |  |  |
| Water | Water | 0 |
|  |  |  |
| Wetlands | Emergent Wetlands | 20 |
|  | Riparian | 10 |
|  | Wetlands | 5 |
