# Supplementary material for: Land Use as a Driver of Patterns of Rodenticide Exposure in Modeled Kit Fox Populations
Source: PLoS One. 2015 Aug 5;10(8):e0133351. doi: 10.1371/journal.pone.0133351 (PMC4564287; doi:10.1371/journal.pone.0133351)
Supplement: S2 Table — (DOCX) [file pone.0133351.s007.docx]

S2 Table. Land-cover classes, SGAR exposure score, and source of map data.

| **Risk value** | **Land cover class** | **Map Source and Description** |
| --- | --- | --- |
| 2^[[1]](#footnote-1)^ | Low-density developed | Farmland Mapping & Monitoring Program (FMMP) [1] "Rural residential" category plus those pixels within FMMP's "Urban" category that were further reclassified (by NLCD) as "developed, low intensity" (See "Urban" category). |
| 2 | Confined Animal Agriculture | Direct from FMMP |
| 2 | Semi-agricultural and Rural Commercial Land | Direct from FMMP: Farmsteads, agricultural storage and packing sheds, unpaved parking areas, composting facilities, equine facilities, firewood lots, and campgrounds. |
| 1*^[[2]](#footnote-2)^*-2^[[3]](#footnote-3)^ | Urban | FMMP "Urban and built up" category was reclassified according to the National land cover dataset (NLCD) [2]. Low-density developed remained in the "low-density developed" category; med- and high-density developed were assigned to "urban." |
| 1*^[[4]](#footnote-4)^* | Orchards and vineyards | See “Farmland” description below. |
| 0*^[[5]](#footnote-5)^* | Natural | FMMP's "Non-agricultural and Natural Vegetation" was reclassified as natural vegetation and other (according to NLCD). FMMP's "Grazing" was also reclassified, and most of this ended up being classed as "Natural" by NLCD. |
| 0^[[6]](#footnote-6)^ | Farmland | First, all of FMMP's farmland categories were combined. "Cropland" from NLCD (from other categories that got reclassified) was included. That category was then split into “Farmland” and “Orchards and vineyards” according to the cropland data layer [3]. |
| 0 | Grazing | From reclassified NLCD |
| 0 | Vacant or Disturbed Land | Direct from FMMP: Open field areas that do not qualify for an agricultural category, mineral and oil extraction areas, off road vehicle areas, electrical substations, channelized canals, and rural freeway interchanges. In addition, lands that were reclassified according to NLCD as "Developed, Open Space” were included: areas with a mixture of some constructed materials, but mostly vegetation in the form of lawn grasses, where impervious surfaces account for less than 20% of total cover. These areas most commonly include large-lot single-family housing units, parks, golf courses, and vegetation planted in developed settings for recreation, erosion control, or aesthetic purposes. |

**References**

1. California Department of Conservation (2011) California Farmland Mapping and Monitoring Program. Available: consrv.ca.gov/dlrp/FMMP/index.htm.

2. Homer C, Huang C, Yang L (2004) Development of a 2001 national landcover database for the United States. Photogramm Eng Remote Sensing 70: 829–840.

3. Boryan C, Yang Z, Mueller R, Craig M (2011) Monitoring US agriculture: The US Department of Agriculture, National agricultural statistics service, cropland data layer program. Geocarto Int.

4. Morzillo AT, Schwartz MD (2011) Landscape characteristics affect animal control by urban residents. Ecosphere 2: art128.

5. McMillin SC, Hosea RC, Finlayson BF, Cypher BL, Mekebri A (2008) Anticoagulant rodenticide exposure in an urban population of the San Joaquin kit fox. In: Timm RM, Madon MB, editors. Proceedings of the 23rd Vertebrate Pest Conference. University of California, Davis. pp. 163–165.

6. Erickson W, Urban D (2004) Potential risks of nine rodenticides to birds and nontarget mammals: a comparative approach. United States Environmental Protection Agency, Washington, D.C.

7. Bartos M, Dao S, Douk D, Falzone S, Gumerlock E, et al. (2012) Use of anticoagulant rodenticides in single-family neighborhoods along an urban-wildland interface in California. Cities Environ 4: Article 12.

8. Lima LL, Salmon TP (2010) Assessing some potential environmental impacts from agricultural anticoagulant uses. In: Timm RM, Fagerstone KA, editors. Proceedings of the 24th Vertebrate Pest Conference. University of California, Davis. pp. 199–203.

9. Cypher B, McMillin S, Westall T, Van Horn Job C, Hosea R, Finlayson, B, et al. (2014) Rodenticide exposure among endangered kit foxes relative to habitat use in an urban landscape. Cities Environ 7: Article 8.

10. Silberhorn EM, Schnable DL, Salmon TP (2006) Ecological risk assessment for use of agricultural rodenticides in California. In: Timm RM, O’Brien JM, editors. Proceedings of the 22nd Vertebrate Pest Conference. University of California, Davis. pp. 458–462.

11. California Department of Pesticide Regulation (2012) Pesticide use database. Available: cdpr.ca.gov. Accessed 8 January 2012.

1. Based on [4–7]. [↑](#footnote-ref-1)
2. Based on [8]. [↑](#footnote-ref-2)
3. Based on [9]. [↑](#footnote-ref-3)
4. Based on [10] as well as low levels of SGAR use reported in orchards [11]. [↑](#footnote-ref-4)
5. Based on [4,5]. [↑](#footnote-ref-5)
6. Based on [10] and very low levels of SGAR use reported in croplands [11]. [↑](#footnote-ref-6)
